# Editorial: Tissue-resident immune cells in tumor immunity and immunotherapy

**DOI:** 10.3389/fcell.2022.1068720

**Published:** 2022-11-22

**Authors:** Annalisa Del Prete, Qi Wu

**Affiliations:** ^1^ Department of Molecular and Translational Medicine, University of Brescia and IRCCS Humanitas Research Hospital, Rozzano-Milan, Italy; ^2^ Tongji University Cancer Center, Shanghai Tenth People’s Hospital, Tongji University School of Medicine, Shanghai, China

**Keywords:** tissue-resident immune cell, macrophage, natural killer cells, dendritic cells, tertiary lymphoid structures, tumor immunity

**FIGURE 1:**
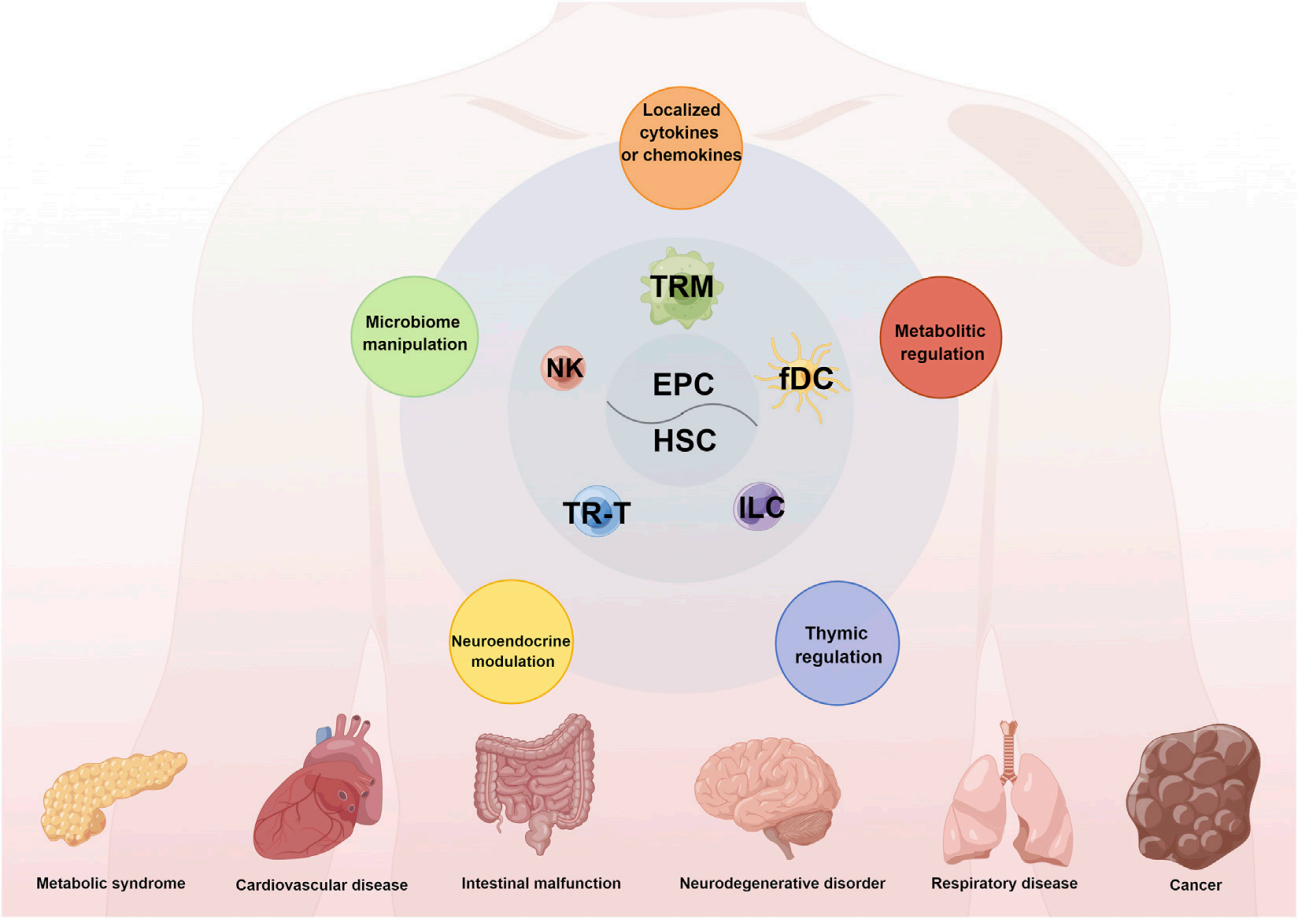
The origins, compositions, and functions of TRICs in human diseases. Tissue-resident immune cells (TRICs) mainly originate from embryonic progenitor cells (HSC) or adult haematopoietic stem cell (HSC). And TRICs mainly consist of diverse tissue-resident macrophages (TRM), NK cell, tissue-resident T (TR-T) lymphocytes, innate lymphoid cells (ILC) and follicular dendritic cells (fDC). TRICs have a vital effect on various human diseases like metabolic syndrome, cardiovascular disease and cancer through the integrated mechanisms including metabolic regulation, thymic regulation, neuroendocrine modulation, microbiome manipulation and secretion of cytokines and chemokines.

Tumor microenvironment (TME) is a complex ecosystem and consists of tumor cells, multiple stromal cells and diverse immune cells (Wu et al., 2021a). From the perspective of immune cells, recent clinical practice reveals that immunotherapies have become remarkable breakthroughs in cancer treatment strategy. Clinical immunotherapies are composed of oncolytic virus, enhanced co-stimulators, vaccines, inhibitory immune checkpoint inhibitors (ICIs) and adoptive cell therapies. As a representative of ICIs, PD1/PD-L1 blockades have been approved for clinical appliance. However, they fail to generate durable responses in most patients. Our understanding of intrinsically and extrinsically immunosuppressive mechanisms for the resistance of ICIs has markedly increased (Wu et al., 2021b). Therefore, the development of strategies to reverse immunosuppressive microenvironment and surmount resistance to immunotherapy have become momentous. Tissue-resident immune cells (TRICs) are immune cells existing in non-immune organs like skin, liver, lung and gastrointestinal tract. They mainly include innate lymphoid cells, macrophages, resident memory T cells, natural killer cells, natural killer T cells, non-classical T cells, and memory B cells (Liu et al.). TRICs are deemed as a bridge that connects innate immunity to adaptive immunity. They enable to remodel the TME and can modulate tumor progression (Liu Z. et al., 2022). However, the potential mechanisms underlying TRICs to host anti-tumor immune response remain unknown. In the present Research Topic, a total of nine articles including two original articles and seven reviews are published to discuss the development, distribution, functionality and therapeutic potential of TRICs in neoplastic disease (Figure 1).

In response to foreign threats or original ontogenesis, these TRICs are capable to resident and adapt to life within diverse tissues. These site-specific immune cell compositions reflect their distinct localization within tissue niches, wherein they form an integral part of the immune sensing network, monitor for local perturbations in homeostasis throughout the body and provide defense against malignancies. For example, Liu et al. characterize the neuroendocrine modulation in tissue-specific immune cells, including regulations through autonomic nervous system, sensory nerves and various neuroendocrine factors. Hence, the involvement of neuroendocrine-tissue specific immunity axis in tumor biology requires to be further explored in the future and may represent a potential target for the immunotherapy of tumors.

Given the tissue-specific structure and location of TRICs, tertiary lymphoid structures (TLS) represent one of the most studied examples of TRIC aggregates. TLS are deemed as organized ectopic lymphoid organs developing in non-lymphoid tissues during chronic inflammation and cancer in response to persistent antigen stimulation (Sautès-Fridman et al., 2016). In Rossi et al., several aspects of tumor-associated TLS are described including their composition, the promoting or inhibitory mechanisms of TLS development, and their prognostic value. High level of heterogeneity has been described in TLS cellular composition, which includes innate and adaptive immune cells such as macrophages, dendritic cells, mast cells, and T and B lymphocytes which are recruited and activated by the inflammatory milieu. Upon prolonged inflammatory stimuli including cancer, TLS can present lymphoid aggregates resembling B cell follicles with germinal centers and T cell areas supported by fibroblastic reticular cells, follicular dendritic cells and high endothelial venules (Siliņa et al., 2018). TLS are the sites where continuous tumor-antigens exposures activate local and systemic T and B cell responses, which control tumor growth, as reported for different tumor types (Dieu-Nosjean et al., 2016). A crucial role is played by the non-hematopoietic stromal component, which include fibroblasts, vascular/lymphatic endothelial cells, and epithelial cells, both in maintaining the architecture of TLS and in shaping the local cross-talk with immune cells by the production of proinflammatory cytokines and chemokines, that drive the recruitment of myeloid and lymphoid cells. Among the myeloid compartment, dendritic cells, as antigen presenting cells, are considered sufficient to TLS induction and maintenance, and are associated with better prognosis in cancer (Sautès-Fridman et al., 2019). Also, macrophages and neutrophils are present on TLS, but their role need to be better elucidated. Finally, the detrimental role of Tregs in TLS is described, with the mechanisms underlying their recruitment and expansion, and the suppressive role of follicular T regs, which are considered potential immunotherapeutic targets for improving the efficacy of ICI therapy. Furthermore, the immunosuppressive landscape induced by Treg was carefully analyzed by Riaz et al. in non-alcoholic fatty liver disease (NAFLD)-associated hepatocellular carcinoma.

Functionally, TRICs can play a dual role since, in the remodeling of the TME, they can foster or suppress tumor response (Liu Z. et al., 2022). For example, the macrophages locating in peritoneum shape a high-energy and chronic inflammatory microenvironment that favors tumor metastasis. Likewise, the peritoneal resident macrophages impair the anti-tumor response of effector T cells and compromise therapeutic efficacy of ICIs (Xia et al.). Similarly, intestinal macrophages contribute to the regeneration of intestinal epithelial cells and the immune homeostasis in intestinal mucosa. These macrophages are capable to polarize into diverse phenotype in response to the endogenous and environmental cues. Ma et al. decipher the origin and mechanisms of maintenance of intestinal macrophages. They also describe the interplays between intestinal macrophages and internal and external stimuli, and highlight the role of intestinal macrophages in the inflammatory bowel disease occurrence and development. In addition, the review by Busà et al. analyze the contribution of innate immune cells, including NK cells, dendritic cells, macrophages, myeloid-derived suppressor cells (MDSCs), and innate lymphoid cells (ILCs), in the modulation of the tumor microenvironment and in shaping the adaptive tumor response.

Immunotherapy has allowed the field of oncology to turn a critical corner. And TRICs maybe major candidates for therapeutic manipulation. As a novel immunometabolic checkpoint, adenosine interacts with its receptors named A1, A2A, A2B, and A3 to promote cancer cell proliferation, neoangiogenesis, immunoescape and metastasis. In TME, adenosine mainly derives from the degradation of ATP, ADP, UTP, and NAD through several ectonucleotidases like CD38, CD39, and CD73. Adenosine not only enable to limit the inflammatory reaction, but also suppress anti-tumor immunosurveillance. Hence, nano-drugs targeting CD39 or CD73 deserve detailed study to increase anti-cancer immune responses (Ferrari et al.). Moreover, Yu et al. generate some novel chimeric antigen receptor (CAR) T cells *via* the construction of tumor-specific cell surface antigen on CAR-T cells. For instance, they create the CAR-T cells overexpressing glucose-regulated protein 78 (GRP78) on cell surface (csGRP78). These engineered CAR-T cells high-efficiently eliminate tumor cells and prolong survival *in vitro* and *in vivo*. In addition, Zhao et al. demonstrate that the use of human or humanized antibody fragments for CAR construction can reduce the immunogenicity of the CAR and improve the therapeutic efficacy. However, these approaches need further clinical investigation. Therefore, developing the therapeutic strategies based on TRICs in the progressive disease is urgently required.

Collectively, this Research Topic describes the origins and biology of TRICs and the function of TRICs in tumorigenesis and malignant progression. It also confirms the importance of TRICs in cancer prevention or treatment and uncovers some unexplored knowledges in the field. In the future, the ontogeny of TRICs especially the key regulators involved in the process should be elaborated. The development of specific druggable targets of TRICs should be further explored and applied in a “personalized medicine” perspective.
